# Integrating Levels of Hierarchical Organization in Porous Organic Molecular Materials

**DOI:** 10.1007/s40820-023-01237-9

**Published:** 2024-01-12

**Authors:** Jesus Ferrando-Soria, Antonio Fernandez

**Affiliations:** 1 Instituto de Ciencia Molecular (ICMol), Universidad de Valencia, 46980 Valencia, Spain; 2 School of Science, Loughborough University, Loughborough, LE11 3TU UK

**Keywords:** Porous organic molecular materials, Hierarchy, Hydrogen-bonded organic frameworks, Porous cages, Fullerene

## Abstract

Porous organic molecular materials (POMMs) are an emergent class of molecular-based materials characterized by the formation of extended porous frameworks, mainly held by non-covalent interactions. POMMs represent a variety of chemical families, such as hydrogen-bonded organic frameworks, porous organic salts, porous organic cages, C − H⋅⋅⋅π microporous crystals, supramolecular organic frameworks, π-organic frameworks, halogen-bonded organic framework, and intrinsically porous molecular materials. In some porous materials such as zeolites and metal organic frameworks, the integration of multiscale has been adopted to build materials with multifunctionality and optimized properties. Therefore, considering the significant role of hierarchy in porous materials and the growing importance of POMMs in the realm of synthetic porous materials, we consider it appropriate to dedicate for the first time a critical review covering both topics. Herein, we will provide a summary of literature examples showcasing hierarchical POMMs, with a focus on their main synthetic approaches, applications, and the advantages brought forth by introducing hierarchy.

## Highlights

- This review covers the extent of the integration of hierarchy in porous organic molecular materials (POMMs) for the first time.
- Three main hierarchies are identified in POMMs: composition, architecture, and porosity.
- The synthesis and applications of hierarchical POMMs, while highlighting the advantages of having hierarchy, are discussed.

## Graphical abstract

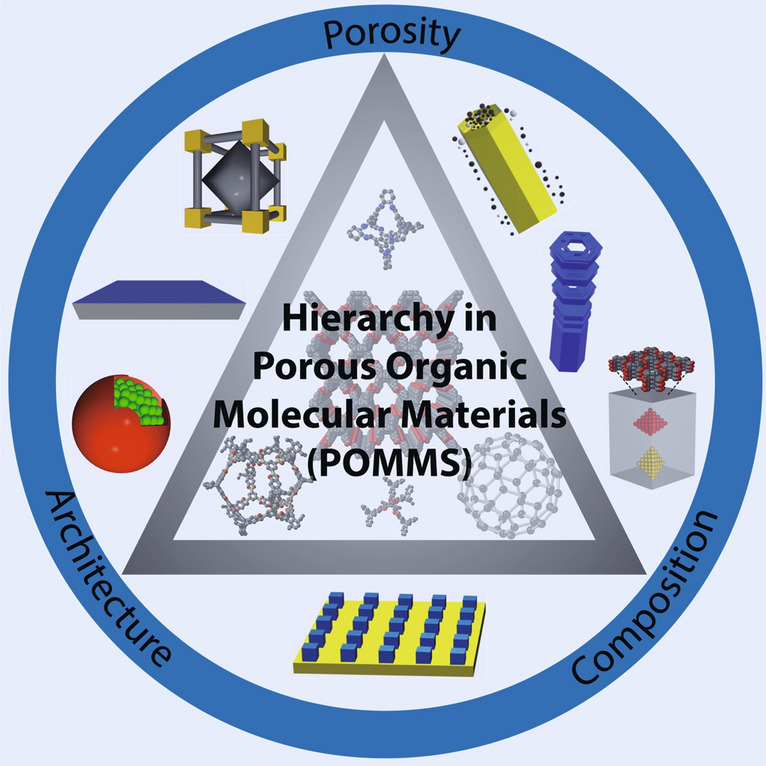

## Introduction

In the realm of porous materials, porous organic molecular materials (POMMs) are an emergent class characterized by the formation of extended porous frameworks, mainly held by non-covalent interactions such as hydrogen bonds, ionic interactions, π–π stacking interactions, among others [1, 2]. From the chemical point of view, POMMs encompass a variety of chemical families, such as hydrogen-bonded organic frameworks (HOFs) [3, 4], porous organic salts [5], porous organic cages (POCs) [6], C–H⋅⋅⋅π microporous crystals [7], supramolecular organic frameworks (SOFs) [8], π-organic frameworks [9–11], halogen-bonded organic framework (XOF) [12], and intrinsically porous molecular materials (IPMs) [13]. Although POMMs are less studied compared to other porous materials, they have already been demonstrated to possess unique properties that are complementary to zeolites [14], covalent organic frameworks (COFs) [15] and metal organic frameworks (MOFs) [16]. High crystallinity and flexibility, low weight and inherent toxicity, good recyclability, great solution processability and self-healing properties, are a unique combination of properties that makes POMMs excellent candidates for a vast range of applications. Another key advantage of POMMs lies in their tunability and diversity. This chemical versatility can be used to design materials with tailored properties for specific applications, leading to enhanced performance and efficiency [17]. Likewise, this also entails the need for a great variety of synthetic strategies and building blocks [1]. This variety of building blocks is translated into a variety of chemistries and properties. For example, a wide range of chemical stabilities in POMMs can be found, with enormous progress being achieved in some POMMs such as HOFs and POCs, to the point of being stable in extreme conditions of pH, boiling water, and strong redox conditions. Another fundamental difference among the families of POMMs is how the porosity is constructed during crystallisation. Materials with intrinsic porosity display pores or voids as integral part of the molecule used as building unit. Therefore, the intrinsic porosity is present before crystallisation and introduced during the molecular design as pre-assembled. In contrast, extrinsic porosity is formed during the crystallization, through the inefficient packing of the molecular precursors. Examples of materials with intrinsic porosity are POCs, constructed by distinct (zero-dimensional or 0D) macromolecules, resulting in materials with inherent voids due to their designed cage shape. During crystallisation, POCs can also form extrinsic porosity through inefficient crystal packing of these macromolecules. In contrast, a characteristic example of materials with extrinsic porosity are HOFs, as a new class of crystalline materials composed of organic molecular precursors linked together through hydrogen bonds, and in most cases π-π stacking interactions, yielding 2D and 3D porous frameworks. As the geometry of the molecular precursor is affecting the final assembly, several aspects should be considered during the design of molecular precursors of POMMs. First, common strategies to other porous materials, such reticular chemistry, are not easily applied to POMMs. This is mainly the difficulty of predicting the crystal packing and hence it is not trivial to design POMMs molecular precursors de novo. That difficulty is exacerbated when additional chemical functionalities are introduced during the synthesis of the molecular precursor. Thus, and despite of being a new field with challenges ahead, POMMs have gained significant attention in recent years and have reached early success in fields of gas separation, catalysis, sensing, drug delivery, and environmental remediation [2].

**Fig. 1:**
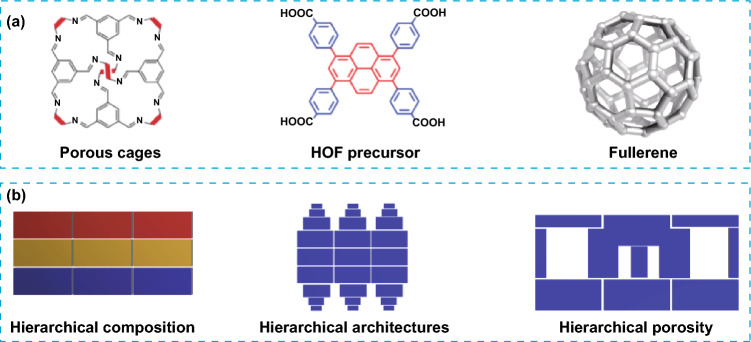
**a** Representation of some of the most common organic building-blocks used for the fabrication of hierarchical POMMs. **b** Schematic illustration of the three main types of hierarchy in porous materials. Creative Commons

Hierarchy is a very fundamental property in many materials [14]. It refers to the presence of multiple levels of organization within a material, ranging from the molecular scale to the micro-, meso- and macroscale. Compared to traditional materials with homogeneous microstructures, where the properties governed by the arrangement of atoms or molecules at a single length scale, hierarchical materials exhibit a complex organization across multiple length scales. Wood and bones are some examples of materials found in nature with hierarchical organization [18]. In these materials, each level contributes to the overall structure and properties, forming interactions and synergies between the different length scales, and leading to properties and performances that are out of reach for most of the materials operating at a single scale [19, 20]. In the field of porous materials, the incorporation of multiscale has been widely adopted in zeolites [21], explored in MOFs [22] and to some extent in COFs [23], allowing to obtain materials with multifunctionality and optimized properties. In POMMs, particularly, the first degree of hierarchy can be considered the crystalline packing which defines the primary porosity and architecture. The next level of hierarchy is introduced at higher scales, by either incorporating secondary architectures, combining micro-, meso or macroporosity, or integrating different materials at different scales. Recent literature has focused on POMMs. A recent review by Little et al. highlights the relevance of POMMs for a wide range of applications [1], where POMMs can be considered as ideal candidates. Similarly, Halliwell et al. focus their review on the synthetic strategies used for the obtention of POMMs with meso- or macroporosity and the combination of multiscale pores [2], and how the porosity affects the properties of these materials. Given the fundamental importance of hierarchy in materials, and the relevance that POMMs are reaching in the field of synthetic porous materials, we consider it appropriate to dedicate, for the first time, an integral overview covering both topics. Herein, we will summarize examples found in the literature of hierarchical POMMs (Fig. 1), focusing on the main synthetic routes and their applications, while trying to underline the advantages of introducing hierarchy. For the sake of clarity, we will divide the sections following the generally accepted classification of hierarchical materials according to their composition, architecture, and porosity [24]. Hierarchical composition refers to the multiscale order of a material having a mixture of 2 or more compositions. Hierarchical architectures have very defined structures at more than one level of organization and hierarchical porosity is present in materials with more than one pore of different scale.

## Hierarchical Composition

**Fig. 2:**
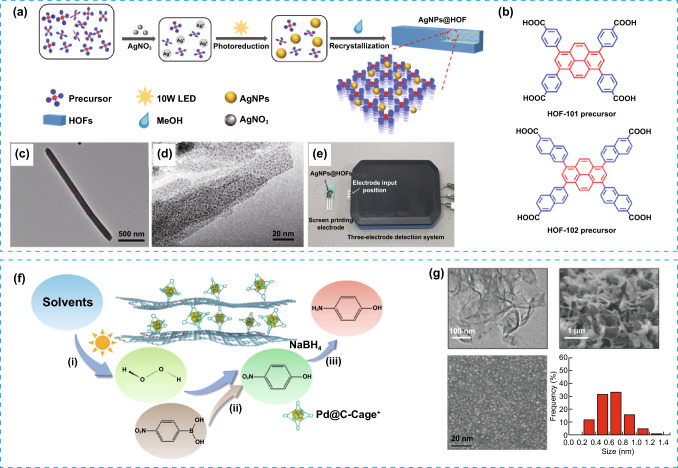
**a** Illustration of the process followed for the synthesis of AgNPs/HOFs and AgNPs@HOFs nanocomposites. **b** Building blocks of HOF-101 and HOF-102. **c**, **d** Images obtained by TEM of HOF-101 and AgNPs@HOF-101, respectively. **e** Image of a three-electrode detection system for CWA detection, fabricated with AgNPs@HOF-101. **f** Illustration of the three-step sequential reaction for the conversion of 4-nitrophenyl boronic acid to 4-amino phenol with Pd@C-Cage^+^/ C_3_N_4_^–^ catalyst. **g** Top from left to right: TEM and SEM images of C-Cage^+^/C3N4^–^. bottom, from left to right: HAADF-STEM image and size distribution of Pd@CCage^+^. Modified with permission of [27, 28]. Copyright Wiley–VCH 2022 and ACS 2022

**Fig. 3:**
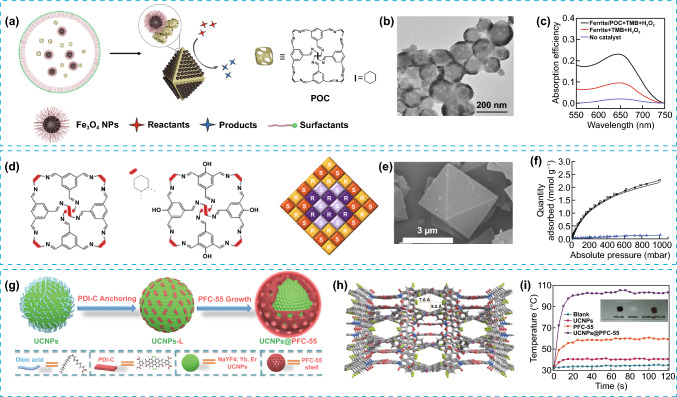
**a** Schematic illustration of co-assembly of Fe_3_O_4_ nanocrystals and POCs molecules. **b** TEM images of hybrid 8.3-nm- Fe_3_O_4_-POC assembly. **c** UV–vis absorption spectra (following the absorption of 3,3′,5,5′-Tetramethylbenzidine (TMB) as chromogenic substrate), showing the catalytic performance of Fe_3_O_4_-POC, in absence or presence of 8.3-nm Fe_3_O_4_ nanocrystal assemblies. **d** Molecular representation of the porous cages for CC3 (left), CC19 (center) and general scheme showing the structure of a core–shell multicomponent heterochiral cage cocrystals (right) (core = purple/mauve; shell = yellow/orange). **e** SEM image of a large CC3-RScore/CC19-RS shell crystal. **f** Gas adsorption (closed symbols) and desorption (open symbols) isotherms for CO_2_ (black squares) and methane (blue triangles) for CC3-RScore/CC19-RS shell crystal. **g** Fabrication of core–shell UCNPs@PFC-55. **h** Crystal packing of PFC-55 porous frameworks, showing the open channels formed through the stacking layers. **i** Comparison curves for the photothermal conversion for UCNPs, PFC-55, UCNP@PFC-55 powders under NIR irradiation. Modified with permission of [29–31]. Copyright Elsevier, Wiley–VCH 2018, 2021 and 2022

The combination of different materials represents an attractive strategy for the integration of complementary, or even in some cases incompatible, properties in a single material, otherwise impossible or at least very challenging to integrate in a single material. Another fundamental consideration is how these materials are arranged in the mixture, as this arrangement greatly alter the composite properties [25, 26]. Hence, a multiscale arrangement represents another variable to consideration during the design of the porous composites. Hierarchical composition refers to a mixture of compositions in a material that are organized at more than one scale. This contrast with hybrid materials, where no order or multiscale order is required. One example of POMMs with multiscale compositional arrangement was reported for the encapsulation of sub-nanometre silver nanoparticles (AgNPs) in multifunctional HOFs (HOF-101 and 102), yielding composites (AgNPs@HOF) with enhanced photoelectrochemical and sensor properties [27]. AgNPs@HOF were prepared from HOF precursors mixed with a solution of AgNO_3_, forming a mix of Ag(I) ions and HOF precursors. AgNPs were then assembled and integrated into HOFs via in situ reduction of the encapsulated Ag(I) ions using light irradiation (Fig. 2a, b). AgNPs@HOF represents an elegant example of the great potential of integrating hierarchical composition in POMMs as sensors, in a selective, sensitive, and rapid manner for the detection of wide range of highly toxic chemical warfare agents (CWA). The synergistic relationship between size exclusion effect by the HOF and the specific chemical recognition between halogen groups in CWA and the Ag in AgNPs@HOF, renders versatile sensors with high selectivity and very low detection limit that can be easily integrated in a portable sensor device (Fig. 2e). This work also represents a clear example of double hierarchy, in composition and architecture, due to dual multiscale structural and compositional organization. In another example, modified porous organic cages (CC3) were used as compartmentalization units for two catalysts, palladium clusters and carbon nitride, to render a hierarchical system (Fig. 2f–g, Pd@C-Cage^+^/C_3_N_4_^–^) [28]. C-Cage^+^ was initially prepared from CC3 cage and added into a solution of C_3_N_4_^–^ during sonication, thus promoting a homogeneous dispersion with strong electrostatic interactions. These two catalysts, although incompatible in homogeneous solution, were both stabilized in presence of CC3, where the palladium clusters were hosted within cationic porous organic cages (Pd@C-Cage^+^) and complexed with anionic carbon nitride (C_3_N_4_^–^). Pd@C-Cage^+^/ C_3_N_4_^–^ can efficiently catalyse complex multistep chemical reactions, including two- and three-steps, as well as convergent catalysis. Mechanistically, it was proposed that the porous organic cages played a prominent multirole in the enhanced catalytic behaviour, mainly stabilization of the palladium clusters that creates substrate channelling effects and compartmentalization of the two catalytic sites. Similarly, porous organic cages following an emulsion-confined strategy can co-assemble with nanoparticles to render a compositional and structural hierarchical material (Fe_3_O_4_-POC) [29]. In particular, this system consists of two-dimensional Fe_3_O_4_ nanoparticle superlattices self-assembled on octahedral porous organic cages colloidal crystals (Fig. 3a, b). The resulting hierarchical material exhibited strong peroxidase-mimic activity for the conversion of 4-nitrophenyl boronic acid to 4-amino phenol in water, resulting in two-times higher catalytic activity than Fe_3_O_4_ nanocrystal alone (Fig. 3c). Notably, this enhanced catalytic activity of Fe_3_O_4_-POC is despite of the hydrophobic nature of the hierarchical assembly, which is covered by a bilayer of aliphatic chains and in contrast with the common nature of reported artificial enzymes with hydrophilic surface. Unexpectedly, the authors also observed the enzymatic activity was dependent on the size of the nanoparticles, with larger sized Fe_3_O_4_ nanocrystal leading to high catalytic activity.

Core–shell nanostructures composed of distinct racemic or quasiracemic porous organic cages represent a nascent field in materials with hierarchical composition [30]. One example of synthesis of such complex systems was obtained by taking advantage of the lower solubility of the racemic or quasiracemic materials, the chiral recognition of enantiomers and the similar lattice parameters for the different porous organic cages, which promoted the epitaxial growth, and consequently, the formation of core–shell structures (Fig. 3d, e). This, for example, allowed to obtain the pair CC3-RScore/CC19-RSshell and CC19-RScore/CC3-RSshell by the sequential addition of solutions of the R and S cage enantiomers by exploiting the chiral recognition, demonstrating that its surface chemistry is governed by the functionality decorating the shell layer. It was also observed a synergistic effect between components in the CC3-RScore/CC19-RSshell system that can be used for gas adsorption applications. The combination of the high CO_2_ sorption capacity by the CC3-RS core along with the CO_2_ selectivity of the CC19-RSshell allowed to achieve high CO_2_ selectively from a CO_2_/CH_4_ gas mixture, rendering a system with enhanced properties compared to the individual cages components (Fig. 3f). The implication of this work is extended beyond porous organic cages, as this strategy could be used to combine porous organic cages with others porous materials such as MOFs or improving the integration of porous organic cages in mixed-matrix membranes. The same core–shell approach to integrate complex hierarchical composition in a material could be extended to components of different nature, rendering materials with synergistic interactions between components and improved functionality. Another great example of this approach is the combination of HOFs and nanoparticles to form core–shell UCNPs@PFC-55 (Fig. 3g). Ostwald ripening-mediated grafting was used to assemble the HOF “shell” via ligand-grafting of oleate-stabilized UCNPs “core” particles. Perylenediimide-based HOF (PFC-55) can maintain a free radical state and show photothermal and photodynamic capacities under visible light [31]. However, it exhibits a weak absorption in the near-infrared (NIR) region, which limits its bio-applications. Thus, the construction of a core–shell hierarchical nanocomposite UCNPs@PFC-55, with upconversion nanoparticles UCNPs at the core and designed overlapping between core emission and shell excitation, represents a powerful approach for the upconversion of NIR light to visible region, which further excite the HOF shell to render an efficient photothermal and photodynamic antimicrobial activity (Fig. 3i).

**Fig. 4:**
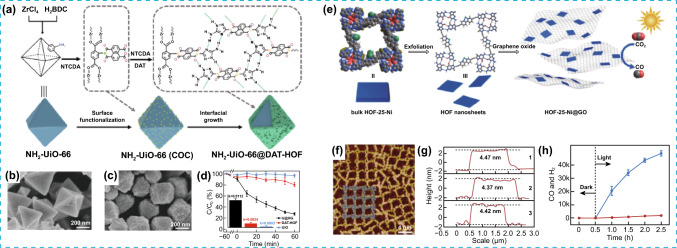
**a** Scheme of the synthetic procedure followed for the obtention of NH_2_-UiO-66@DAT-HOF. **b**, **c** SEM images of NH_2_-UiO-66 precursor and NH_2_-UiO-66 MOF@DAT-HOF hybrid, respectively. **d** Comparsion of the photodegradation efficiencies of tetracycline and the apparent reaction rate constants (inset) between NH_2_-UiO-66 MOF@DAT-HOF hybrid and different photocatalysts. **e** Schematic synthesis of HOF-25-Ni nanosheets and HOF-25-Ni@GO for the photocatalytic conversion of CO_2_ to CO. C: grey; N: cyan; O: red; H: white; Ni: green; **f** STM images of HOF-25-Ni. **g** Height profile distribution of three measured random HOF-25-Ni nanosheets. **h** Time-dependent CO and H_2_ evolution for the photocatalytic reduction of CO_2_ under visible light irradiation in presence of HOF-25-Ni@GO-10. Modified with permission of [32, 33]. Copyright Wiley–VCH 2022

This core–shell approach has also been extended to the combination of HOFs and MOFs [32]. The archetypal NH_2_-UiO-66 MOF, characterized by its high stability, was used as core unit, and functionalized with naphthalenetetracarboxylic dianhydride, precursor of the DAT-HOF, leading to the formation of the nanocomposite NH_2_-UiO-66 MOF@DAT-HOF (Fig. 4a–c). The synthesis was possible through the functionalisation of the MOF forming the core, and the posterior interfacial growth HOF (DAT-HOF) shell on NH_2_-UiO-66 MOF. The resulting hierarchical material exhibited an improvement on its structural and photochemical stability—up to eight cyclic runs—, as well as in the photocatalytic degradation of tetracycline, compared to the isolated constituents. This stability was attributed to the hierarchical nature of the material, with a core–shell structure and synergistic interaction between components, which also extended the utilization range of the visible light and improved the charges separation (Fig. 4d). This enhanced functionality was also extended to the degradation of other emergent contaminants, such as antimicrobials and pesticides. Ultrathin HOF nanosheets (HOF-25-Ni) were prepared in high yield by post-synthetic metalation of a robust guanine-quadruplex HOF precursor with Ni(ClO_4_)_2_.6H_2_O, followed by solution-supported sonication exfoliation methodology (Fig. 4e–g) [33]. The high yield obtained during the preparation of HOF-25-Ni was attributed to the intrinsically preferred exfoliation nature of the selected HOF along with the post-synthetic metalation with nickel(II) ions. HOF-25-Ni was then dispersed on graphene oxide (HOF-25-Ni@GO) and tested as catalyst, exhibiting an efficient activity for the visible-light-driven CO_2_ reduction reaction –assisted with [Ru(bipyridine)_3_]^2+^ and triisopropanolamine–, showing a high conversion rate and 96.3% CO selectivity (Fig. 4h).

**Fig. 5:**
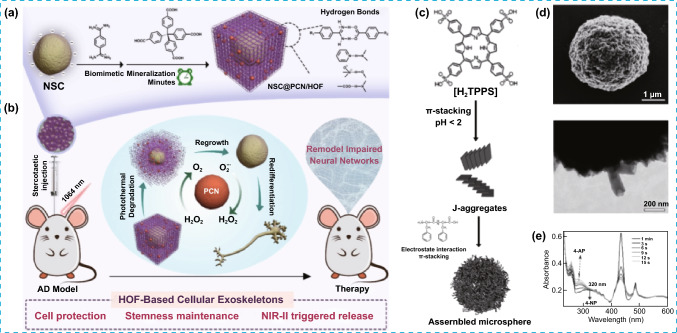
**a** Illustration representing of the formation of composite NSC@PCN/HOF by the encapsulation of neural stem cells within a HOF. **b** Process for the remodeling of impaired neural networks in mice model. **c** Proposed mechanism for the self-assembly of peptide–porphyrin microspheres, involving dipeptide-mediated charge screening of the porphyrin J-aggregates in combination with local stacking of dipeptide cations specifically around the J-aggregates. **d** SEM (Top) and TEM (bottom) images of a single microsphere showing irregular surface texture and the aggregated nanorods, respectively. **e** UV/Vis spectra of the evolution of the peptide–porphyrin assembly indicated by the variations in the intensities of peaks at 434 and 490 nm Modified with permission of [34, 35]. Copyright Wiley–VCH 2014 and 2022

HOFs can also be valuable candidates to develop hierarchical biocomposites by encapsulating large assemblies. For example, the encapsulation of neural stem cells (NSC) within a HOF doped with porous carbon nanospheres (PCN) provides a robust artificial exoskeleton with hierarchical hydrogen bonds and oxidative stress resistance –with catalase and superoxide dismutase activities–, and NIR-II photodegradable nature, which circumvent some of the major drawbacks found in transplantation of neural stem cells (NSC@PCN/HOF, Fig. 5a) [34]. The biocomposite was assembled by adding a solution of HOF precursors followed by the sequential addition of PCN and NSC, where the NSC are stabilized by the strong electrostatic interactions with the HOF framework. In this study, it was observed that the multifunctional nature of the final bio-composite resulted from the hierarchical composition and synergistic interactions between components. This approach has been further implemented, taking advantage of the porous nature of carbon nanospheres to charge them with drug molecules –retinol acid– with direct influence on the differentiation of neural stem cells to neurons. The stereotactical transplantation of the prepared hierarchically complex biomaterial into the hippocampus of mice results in an improvement of neural stem cells viability and a significant improvement in memory functions of Alzheimer´s disease mice model, as consequence of promotion of neurogenesis and relieve of cognitive disorders (Fig. 5b). Another interesting example of hierarchical porous composite was obtained during the electrostatically induced co-assembly in water of small biomolecules, such as simple dipeptides and porphyrins, leading to the formation of photocatalytically active, multi-chambered microspheres [35]. The microspheres were synthesized in a sequential manner, mainly driven by π–π stacking and electrostatic interactions, in very acidic conditions within 1 h of mixing (Fig. 5c). These microspheres are porous and possess a water-filled multi-chambered interior, accessible to guest molecules, and constituted by an interconnected network of peptide-porphyrin nanorods, presenting stacks of porphyrins (J-aggregate) with dipeptides interacting electrostatically with light-harvesting abilities. As consequence of the hierarchical structure, this material was used for the sequestration of cationic organic molecules and photocatalysis, promoting the light-induced oxidation of iodide to tri-iodide, as well as the reduction of metal salts and small organic molecules.

## Hierarchical Architectures

**Fig. 6:**
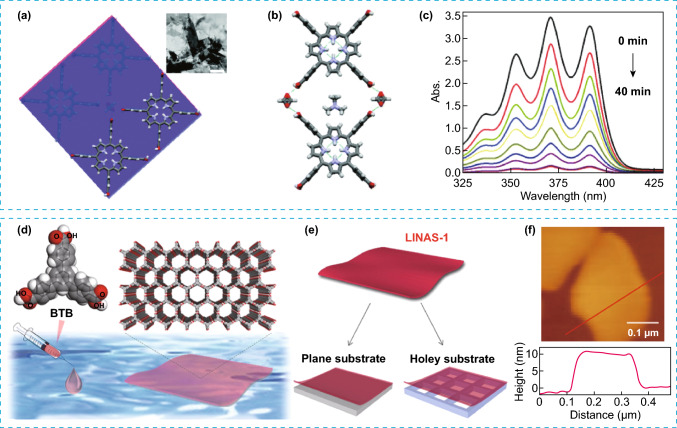
**a-b** Representation of the crystal packing of HOF (SEU-1) showing the TCPP molecules glued by formate yielding 2D square-like grid. Inset: TEM image of SEU-1. Scale bar: 500 nm. **c** Variation of the UV–vis spectra during the photocatalytic degradation of DPA in presence of SEU-1 nanosheets. **d**, **e** Representation of the process for the interfacial synthesis of triphenylbenzene derivative on water, resulting in the creation of crystalline porous nanosheets (LINAS-1) that can be transferred to planar and non-planar substrates. **f** Top: Direct AFM image of LINAS-1 and Bottom: the obtained height profile along the marked red line. Modified with permission of [38, 42]. Copyright 2019 and 2021, RSC and ACS

The integration of architectures at different scales, by combining two or more structural levels [24], one at molecular and a higher up level, could render materials with synergistic or fine-tuned properties by combining the attributes of the individual components. A common challenge during the formation of multiscale architectures is the need of some degree of control during the anisotropic growth, where the orthogonal orientation of the different intermolecular interactions such as hydrogen bonding and π–π stacking present in some POMMs, is proven advantageous during growth. Although obtaining hierarchical architectures in POMMs is a new concept, some initial success was already demonstrated for several families of POMMs. Indeed, examples of superstructures with different dimensionality, scale levels and architectures can be found as thin films, nanosheets, and hollow architectures [36, 37]. Regarding the synthetic strategies used to obtain multiscale architectures, solution processable approaches are commonly employed while introducing or preserving the porosity. For example, atomically thin 2D nanosheets of a highly crystalline HOF (SEU-1) with uniformly cubic morphology was obtained by exfoliation using ultrasonic force-assisted liquid exfoliation technology [38]. SEU-1 consists of TCPP molecules, linked by formate, forming 2D square-like grid skeleton with excellent stability and permanent porosity (Fig. 6a, b). The photocatalytic activity of these 2D nanosheets for the removal of contaminants was tested, showing an increased photocatalytic rate in aqueous systems compared to other HOFs, mainly due to an increased surface area (Fig. 6c) [39–41].

**Fig. 7:**
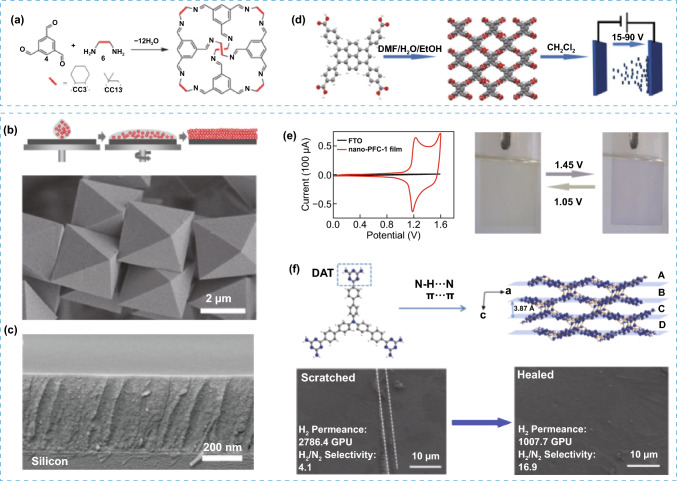
**a** Representation of the synthesis of porous organic cages CC3 and CC13. **b** Top: Example of the solution-process followed for the formation of thin films using organic cages and bottom: SEM image of CC3 crystals. **c** SEM image of the spin-coated film of CC3 on silica support. **d** Representation of process for the fabrication of the nano-PFC-1 film by EPD method. **e** Left: CV curves for nano-PFC-1 film. Right: images of the formed nano-PFC-1 film showing the change in color when a variation of voltage is applied. **f** Top: Representation of the HOF building block precursor and crystal packing of UPC-HOF-6 (C gray, H white, N blue). Bottom: SEM image for the damaged UPC-HOF-6 membrane and the same membrane after healing. Modified with permission of [44, 51, 52]. Copyright Wiley–VCH and ACS 2016 and 2020

Although during the formation of 2D architectures in POMMs, the most common strategy has been the exfoliation, in some cases, different approaches need to be considered. One of these alternative approaches to create 2D assemblies is the interfacial synthesis. For instance, air/liquid interfacial synthetic route was used to assemble a HOF based on triphenylbenzene derivative (LINAS-1) into perfectly oriented highly crystalline non-covalent bonded organic nanosheets [42], while suppressing the favoured assembly of complex interpenetrated structure that is obtained during the synthesis of the bulk crystals (Fig. 6d). The high stability of the nanosheets was key to maintain the crystallinity and pore orientation during their transfer to common substrates such as silicon, quartz, gold, graphite (Fig. 6e). Gas adsorption measurements indicated low affinity to water vapour, suggesting hydrophobic pore environment, and high affinity towards non-polar molecular such as O_2_, which suggests that LINAS-1 could have potential in industrial relevant environments for gas separations. Thin films were also demonstrated for POMMs by spin-coating, where the first example was reported for porous organic cages for sensing applications [43]. Defect-free microporous thin-film were later obtained by solution processable methods for CC3 and CC13 porous cages and tested as membranes for gas separations of mixtures with selectivities of up to 155 for H_2_/CH_4_ and 87 for H_2_/N_2_ (Fig. 7a–c) [44]. Further studies also confirmed the potential of porous cages as films for molecular separations [45–50]. HOFs can also be deposited on surface as thin films for different methods. One method, electrophoretic deposition (EPD), was used to prepare a HOF film (nano-PFC-1) with a reversible electrochromic [51] change from yellow to blue-violet. The film showed high performance with low power consumption, long cycle life, and easy regeneration (Fig. 7d, e). Moreover, post-synthetic modification of the HOF films with redox-active species generated multistate electrochromic behaviour with successive colour changes. As example, the modified film was adsorbed with Fe^2+^ species showing reversible redox peaks and successive colour changes during the CV process, thus demonstrating its potential as material for electrochromic applications. In another example, films with large areas of a HOF (UPC-HOF-6) also were obtained by casting of a solution of DAT precursor on alumina substrates (Fig. 7f). The film assembly was directed by N–H..N and π-π stacking interactions, demonstrating self-healing properties and pressure-responsive performance for gas separation of H_2_/N_2_ mixtures with good selectivity [52].

**Fig. 8:**
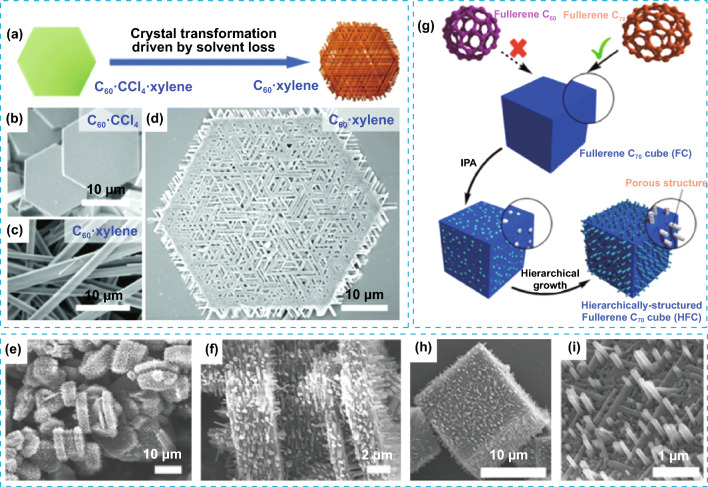
**a** Schematic representation for the solvent loss driven conversion of C_60_ plate to mesh network. SEM images for **b** initial C_60_ plates, **c** synthesized rods and **d** C_60_ mesh network. **e**, **f** SEM images of the mesoporous C_60_ crystals decorated with vertical nanorods. **g** Schematic illustration of the hierarchical assembly of fullerene C_70_ into mesoporous cubes with cube-shaped geometry. **h**, **i** SEM images of mesoporous cubes decorated with nanorods and detailed imaged of the nanocubes. Modified with permission of [53, 54]. Copyright 2016 and 2019, ACS and RSC

Different hierarchical architectures, such as hexagonal mesh networks consisting of nanorods and 2D nanoplates, can be synthesized with fullerene C_60_ by a simple co-solvent inclusion strategy. During the mesh network formation, as result of the conversion of 2D fullerene plates to hcp rods, macroporosity was induced during the structural changes as consequence of the loss of solvent mixture (Fig. 8a–f). This also resulted in the epitaxial growth of ordered C_60_ nanorod arrays, forming out-of-plane vertical rods on the mesh networks [53]. Cubic based micrometric architectures (HFC) of fullerene C_70_ decorated with vertical nanorods can be obtained by ultrasound-assisted liquid–liquid interfacial precipitation (ULLIP). During the growth of the superstructures, mesoporosity of average of 8 nm is introduced, which is translated in a much larger BET surface area compared to the original C_70_ crystals (Fig. 8g–i). These fullerene-based superstructures with high surface are good candidates as materials for the selective detection of aromatic solvent vapours using a quartz crystal microbalance (QCM). Quartz crystal microbalance experiments demonstrated that HFC architecture can act as selective sensor for aromatic guest molecules, mainly due to the high surface area and the hierarchical architecture containing nanorods, that favours the diffusion of aromatic vapours into the mesoporous architectures and the subsequent strong π–π interactions between aromatic groups [54].

**Fig. 9:**
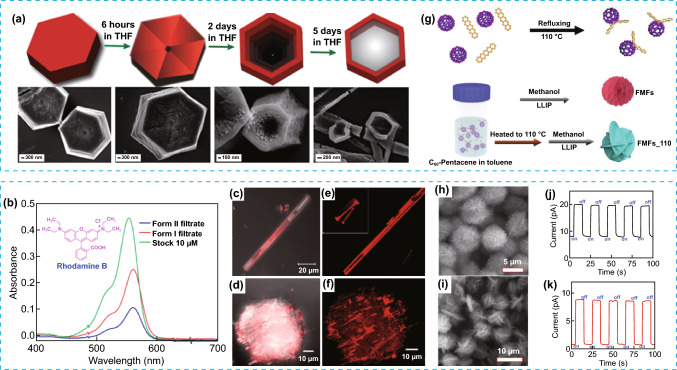
**a** Top: Schematic illustration of the expected mechanism of formation of hollow hexagonal tubes and bottom: FESEM images of evolution during each intermediate step. **b** UV − vis absorption spectra of Rhodamine after adsorption by Form II and Form I. **c**, **d** Optical and confocal laser microscopy images of hollow Form II after RhB dye adsorption and **e**, **f** for Form I. **g** Schematic representation of the synthesis of the FMFs and FMFs_110 by liquid–liquid interfacial precipitation (LLIP) process. **h**, **i** SEM images of FMFs and FMFs_110, respectively. **j**, **k** Time-resolved photoresponse for FMFs and FMFs_110, respectively. Modified with permission of [55, 56]. Copyright 2021 and 2020, Wiley–VCH and ACS

Beyond 2D constructions, another type of hierarchical architecture that can be obtained in POMMs are hollow structures that can be attractive as encapsulation vessels among other applications. One example is the hollow single crystals of a HOF (Form II) prepared from a simple building block, trimesic acid, and obtained by crystallization through intermediate steps involving several morphologies and solid morphology (Form I) [55] as starting point (Fig. 9a). The resulting hollow hexagonal crystalline tubes of Form II were tested for the adsorption and removal of common pollutants and demonstrated being considerable more effective for the Rhodamine B dye adsorption (82 vs 39%) than the solid form of the crystal Form I, mainly due to additional adsorption into the hollow cavity (Fig. 9b–f).

More exotic superstructures such as mesoporous microflowers can be obtained with C_60_-pentacene as building block using liquid–liquid interfacial precipitation (LLIP) [56]. The nanofeatured microflower density, introduced (FMFs and FMFs_110) (Fig. 9g–i) during the hierarchical formation, was controlled by the temperature used during synthesis and proved to be determinant on the performance of these superstructures as photodetectors. Films made of nanofeatured microflowers showed an increase in the current density as the nanofeature density was also increased in the microflowers (FMFs vs FMFs_110), even in absence of illumination. In contrast, films made of smooth microflowers did not show any photocurrent, indicating the importance of multiscale features and how affect the energy transfer (Fig. 9j–k). Both results clearly demonstrated the importance of the presence of structural hierarchy during photo-response, and how it can be tuned by changing the nanofeature density. This tuneability is an attractive feature for these materials and their use on the fabrication of novel optoelectronic devices based on fullerene superstructures.

**Fig. 10:**
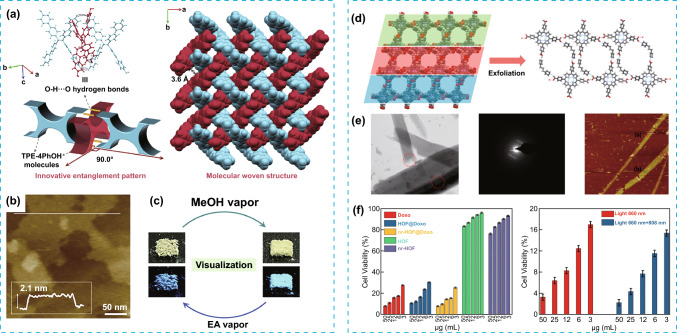
**a** Illustration of the interlocking motif in 2D-90 and the molecular woven structure of 2D-90 obtained by SCXRD. **b** AFM images of exfoliated 2D-90 single crystals. **c** Images of the colour change transformations by dynamic luminescence behaviours under visible light (top) and under 365 nm (bottom). **d** Representation of the crystal packing of the HOF TCPP-1,3-DPP and its exfoliation to obtain 1D Nanoribbons. **e** Left to right: TEM, SAED and AFM image of 1D nanoribbons. **f** Left: In vitro cytotoxicity of Doxo, HOF@ Doxo, nr-HOF@ Doxo, HOF, nr-HOF and for nr-HOF@ Doxo (right) at different concentrations in presence of A549 cells. Modified with permission of [57, 58]. Copyright 2019 and 2021, ACS and Elsevier

The combination of multifunctionality and porosity in POMMs can also be achieved with the goal of extending the range of applications of these materials. For example, a HOF with 2D architecture (2D-90) can be obtained by self-assembly of designed 1D strands with woven architecture, exhibiting large-scale elasticity and reversible structural transformations [57] (Fig. 10a–c). The dynamic molecular woven structure also shows multimode stimuli-responsive luminescence with high-contrast emission colour switching. These properties can be useful in several applications such as sensing, data recording and biomedicine. Additionally, multifunctionality was also demonstrated in atomically thin 1D porous nanoribbons (nr-HOF) prepared by the ultrasonic force-assisted exfoliation [58] of 3D HOF crystals based on TCPP building blocks (TCPP-1,3-DPP). The obtained nr-HOF nanoribbons were tested as drug carriers, demonstrating a high load capacity due to their high surface area and better biocompatibility. The doxorubicin loaded nr-HOF showed higher effectiveness than the pure form of the commercial drug, achieving cell viabilities as low as 1.3% during chemotherapy–photodynamic therapy–photothermal therapy (Fig. 10d–f).

## Hierarchical Porosity

**Fig. 11:**
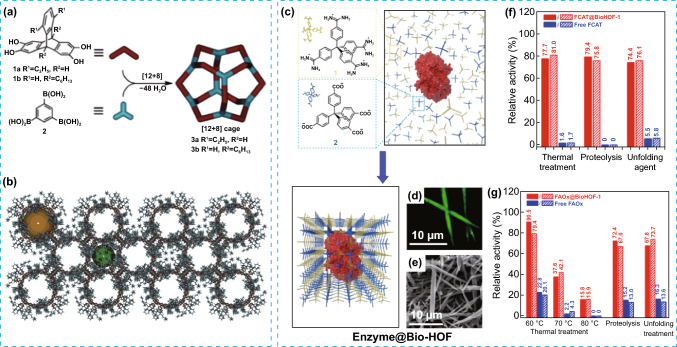
**a** Schematic representation of the synthesis of [12 + 8] boronic ester cages 3a and 3b and formation of the catenated cage 4 during crystallization. **b** Representation of the crystal packing of 4, resulting for 3b, showing the micro and mesoporosity. **c** Representation of the synthesis enzyme@BioHOF-1. **d**, **e** Confocal laser microscopy and SEM images of the resulting FCAT@BioHOF-1 composite, respectively. **f**, **g** Comparison between the relative activity of free FCAT, FAOx, FCAT@BioHOF-1 and FAOx:BioHOF-1 after: thermal heating, exposure to proteolytic trypsin and unfolding agents such as urea. Modified with permission of [60, 61]. Copyright 2014 and 2019, Wiley–VCH and ACS

Multiscale porosity is particularly sought in materials with an inherent tendency to form micropores [26]. Certainly, by accommodating different levels of porosity in a harmonized fashion, new functionalities and improved performance can be achieved, principally when an enhanced mass diffusion, by improving interconnectivity between pores, is required [59]. Several examples of accommodating micro and mesoporosity can be found in different families of POMMs. Two early examples of micro and mesoporosity in single crystals were obtained during the formation of multiple boronic ester bonds from the reaction of 12 triptycene tetraol and 8 triboronic acid molecules, yielding a crystalline framework (4) with catenated cages (Fig. 11a, b). The catenation is held by attractive van der Waals forces and dominated by π–π interactions [60]. Despite of the interlocked nature, the micro and mesoporous packing reveals a relatively high BET surface area (1540 m^2^ g^−1^). In another example, mesoporosity was introduced in a microporous HOF (BioHOF-1) in presence of a template, the nanoscopic enzyme (BSA), during the framework assembly. BioHOF-1, which is composed of water-soluble tetra-amidinium (1) and tetracarboxylate building blocks (2, Fig. 11c), can encapsulate and stabilize relevant enzymes, where the biomolecules also play the role of template during the framework assembly. The biocompatible framework allows the stabilization and protection of enzymes from harsh environments, thus extending their operable pH and temperature range of enzyme activity. Examples of enzymes encapsulated are the fluorescein-tagged catalase (FCAT) and fluorescein-tagged alcohol oxidase (FAOx), resulting in crystalline composites with significantly higher chemical and thermal stability compared to the free enzymes (Fig. 11f–g) [61]. This work is a good example of integrating hierarchical porosity and composition in a single material.

**Fig. 12:**
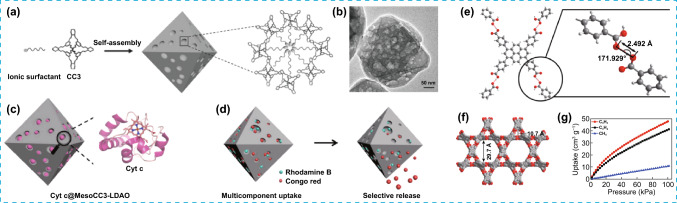
**a** Schematic mechanism for the assembly of MesoCC3 from ionic surfactant molecules and microporous CC3 cages. **b** SEM image of MesoCC3 particle. **c** Representation of Cyt c@MesoCC3-LDAO. **d** Electrostatically gated MesoCC3 for the selective release of Rhodamine B and Congo red. **e** Representation of the hydrogen bonding between carboxylic units. **f** Crystal packing of PFC-2 defining the micro and mesopores. g Adsorption isotherms for CH_4_, C_2_H_2_, and C_2_H_4_ of PFC-2. Modified with permission of [62, 63]. Copyright 2019 and 2021, Wiley–VCH and ACS

Further illustration of the potential of integrating mesoporosity in microporous organic cages for biological applications was exemplified with CC3 cages, yielding micro- and mesoporous MesoCC3. In this case, the mesoporosity was introduced during crystallization via partial accommodation of surfactants within micropores, thus preventing the dense packing during crystal growth due to the size of the surfactant (Fig. 12a). The resulting mesoporosity was advantageous for the immobilization of enzymes such as Cyt c by electrostatic interaction yielding Cyt c@MesoCC3-LDAO, while the combination of micro- and mesoporosity in MesoCC3 serves as drug delivery mechanism based on pH-selective electrostatic gating function incorporated by the surfactants with ionic head (Fig. 12d) [62].

In HOFs, micro- and mesoporosity can also be integrated during framework formation by choosing large and rigid molecular subunits, such as pyrene and benzene core, decorated with COOH groups. For example, the crystallization of the molecular unit formed by a pyrene core and 4 benzoic groups yielded a 3D framework (PFC-2) stabilized by COOH-COOH hydrogen bonds and strong π–π interactions. Gas adsorption applications of industrial relevant mixtures were studied for this HOF, showing a highly selective adsorption of acetylene and ethylene versus methane at room temperature. The high gas uptake, along with the gas selectivity, was rationalized by the highly accessible void space, due to micro- and mesoporosity, and the unpaired hydrogen bond acceptor C=O groups in PFC-2, which significantly increased the affinity between gas molecules and frameworks, resulting in higher selectivity towards acetylene and ethylene hydrocarbons than methane [63].

**Fig. 13:**
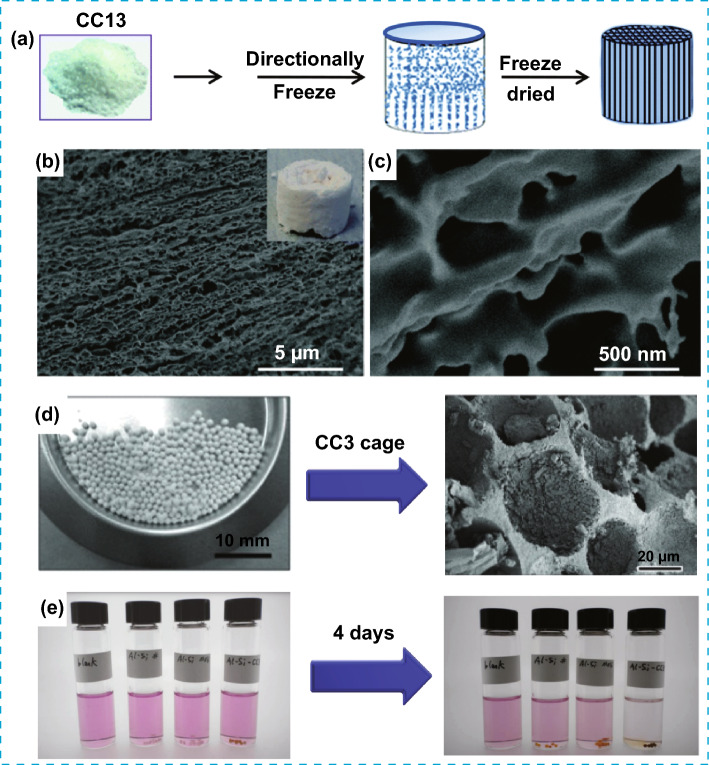
**a** Representation of the preparation of aligned porous monolith from CC13 cages. **b** and **c** SEM images of the monolith at different scale. **d** Left: Images of the macroporous silica beads used as templates and right: SEM image of the internal cross section of the resulting material after the removal of the porous beads. **e** Left: Images of initial iodine solutions with (left to right labels): blank, Al-Si beads equalized by number, Al-Si beads equalized by mass, Cage loaded Al-Si beads. Intervale of time between images is 4 days. Modified with permission [64, 65]. Copyright 2012 and 2015, Wiley–VCH and RSC

The solution processability in POMMs is very advantageous for the introduction of macroporosity in inherently microporous materials. This was demonstrated with solution processable porous organic cages (CC3) used for the formation of monolithic scale materials with aligned micro- and macropores and high interconnectivity, obtained by controlled freeze-drying method (Fig. 13a–c). Compared to conventional monoliths, highly aligned hierarchical porous monoliths can be used as monolithic catalytic support in continuous flow reactions, giving an increased rate of liquid absorption as result of a reduced pressure drop [64]. In another example, the introduction of macroporosity was developed for single component and for racemic mixtures of microporous cages CC3(S,R) coated on inorganic templates (Fig. 13d). The resulting hierarchical material was tested for environmental applications, such as the capture and storage of radioactive forms of iodine produced in the nuclear industry. The cage loaded with inorganic beads was 4.5 times more effective than non-porous beads during the removal of iodine that is from solution [65]. This example further demonstrated that the introduction of macroporosity is commonly advantageous to avoid blocking flow during liquid adsorption for the removal of impurities of liquids such as water and organic solvents required in environmental applications.

**Fig. 14:**
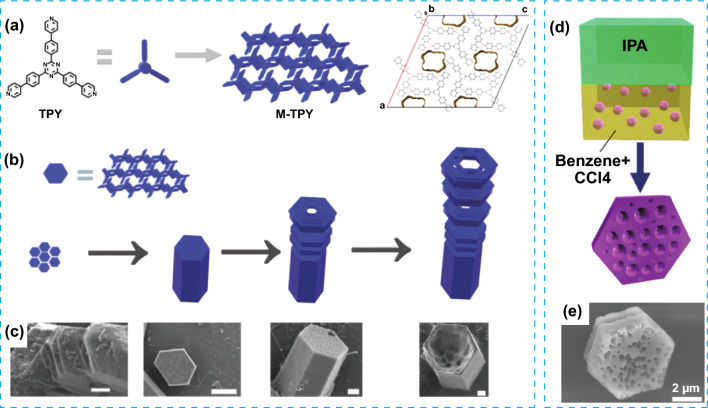
**a** Illustration of the chemical structure of the MM-TPY building block and its crystal packing, showing the microporosity. **b** Top: representation of the platelets as hexagonal blue blocks. Bottom: the proposed mechanism of growth during the formation of MM-TPY crystals, rendering a hollow morphology with hierarchical macroporosity. **c** SEM images of MM-TPY crystals in each stage during crystal growth. Scale bars (left to right): 1, 10, 2, 2 μm. **d** Representation of the LLIP method used for the C_60_ crystallization of meso and microporous C_60_ crystals. **e** SEM image of the resulting C_60_ crystals. Modified with permission [66, 67]. Copyright 2013 and 2022, Wiley–VCH and ACS

HOFs are also compatible with the integration of macroporosity. Micro- and macroporosity was introduced in a tripyridine-based HOF (MM-TPY) during crystallization and under conditions that promoted skeletal growth (Fig. 14a). The difference in the strength and orientation between intermolecular interactions resulted in a highly anisotropic growth rate during the crystal formation, leading to the formation of gaps or voids, defined as microporosity, in the microporous assembly (Fig. 14b, c). Having multilevel porosity could be advantageous in new applications where multiple adsorptions are sought. For example, MM-TPY was tested for the dual adsorption of molecular species in solution and the selective recognition of microparticles. Phenol Red was selectively adsorbed over Methylene Blue within the micropores, while carbon particles were selectively attached within the macropores, mainly due to hydrophobic effects [66].

Combining meso- or macroporosity with microporosity can be relatively easier to obtain in POMMs than combining both meso- and macroporosity. This is due to the stability constraints that could arise when a high density of large pores is present in a framework mainly held by weak intermolecular interactions. The only example reported thus far of meso- and macroporosity in POMMs was achieved in C_60_ fullerene hexagonal crystals and synthesized by liquid–liquid interfacial precipitation process [67]. By adjusting the solvent ratio, the porosity size and geometry could be controlled during the slow evaporation process leading to the removal of entrapped solvent molecules (Fig. 11b–e). The introduction of hierarchical porosity in C_60_ crystals influenced the electrochemical properties of these materials, as indicated by the electrochemical active surface area in these C_60_ crystals, exhibiting higher current density compared to C_60_ crystals with no porosity. The effect of the introducing porosity in porous crystalline material based on C_60_ has clear potential for their use as materials for the fabrication of nanodevices such as organic solar cells and miniaturized organic electronic devices [68].

## Conclusions

The relevance that hierarchy has achieved in the field of porous materials emphasizes the importance of understanding and utilizing hierarchical structures to devise materials with enhanced properties and optimized performance. Given the growing interest for POMMs in the literature, this review aims to cover the extend of the integration of hierarchy in POMMs and thus contribute to the understanding and future advancement of these materials. From the examples reviewed here, we can conclude that the integration of multiscale in POMMs also offers a fascinating avenue for researchers to explore new frontiers in material design and application.

Next clear steps in the field would be the combination of hierarchies, so multifunctionality could be incorporated, and some early examples have been included here. Also, it is expected that certain combination of hierarchies in one material will be particularly challenging, such as the combination of hierarchical porosity with other hierarchies, and the development of purely organic core–shell crystals combining families of POMMs. Additionally, a clearer understanding of the underlying mechanism governing multiscale growth should be pursued, as it remains elusive in most families, along with more detailed studies for the structure–property relationship at different scales. Future avenues to pursue could be the development of applications using solution processable techniques for the integration of POMMs in electronic devices is in its infancy. For example, despite of their potential, the integration of POMMs in electronic devices is still in its infancy. In this case, additional properties in POMMs such as flexibility and elasticity would be an advantage during their integration in flexible electronic devices. Moreover, with their relatively lower toxicity compared to MOFs, simple synthesis, and solution processability, POMMs also seem more logic candidates for biomedical applications, including biocatalysis, and biosensors. However, and despite of the inherent lower toxicity of POMMs, to the author’s knowledge, no studies on the biodegradability of POMMs in relevant environments have been published, which will be required for biomedical and environmental applications.

For future prospective industrial applications, several considerations should be made. Firstly, the inherent solution processability of POMMs [1] represents a key advantage over other porous materials in terms of materials processing and secondly, the study of mechanical properties should be carefully studied if these materials are going to be considered a replacement for current existing materials, or when new applications are targeted. The combination of properties such as flexibility, better biocompatibility, and self-healing nature [52] in a single synthetic material are unique and could be the starting point for the development of novel devices based on these properties. However, a long-standing challenge that needs to be addressed in these materials is the incorporation of pore functionalities, since the inclusion of additional chemical functionalities has profound effects on the crystal structure, in most cases altering the packing completely. The integration of hierarchical composition could partially solve this challenge by designing novel composites with materials that can more easily integrate chemical functionalities.

In a personal note, we are convinced that in the future, POMMs can reach a similar level of development to other porous materials with extended networks such as MOFs and COFs. Clearly, the level of integration of multiscale design among the families of POMMs is very heterogeneous, and although a general synthetic strategy for incorporating hierarchy in all POMM families is difficult to foresee, this review aims to stimulate cross-pollination among different families. With this in mind, we believe that chemical strategies could be shared and serve of inspiration for the construction of more complex multiscale structures that would expand the library of POMMs materials [68].
